# Review on Eye-Hand Span in Sight-Reading of Music

**DOI:** 10.16910/jemr.14.4.4

**Published:** 2021-11-11

**Authors:** Joris Perra, Bénédicte Poulin-Charronnat, Thierry Baccino, Véronique Drai-Zerbib

**Affiliations:** LEAD, Université Bourgogne Franche-Comté, Dijon, France; Université Paris 8, Paris, France

**Keywords:** eye-hand span, eye movements, music reading, expertise, parafoveal processing, multimodal processing

## Abstract

In a sight-reading task, the position of the eyes on the score is generally further ahead than the note being produced by the instrument. This anticipation allows musicians to identify the upcoming notes and possible difficulties and to plan their gestures accordingly. The eye-hand span (EHS) corresponds to this offset between the eye and the hand and measures the distance or latency between an eye fixation on the score and the production of the note on the instrument. While EHS is mostly quite short, the variation in its size can depend on multiple factors. EHS increases in line with the musician's expertise level, diminishes as a function of the complexity of the score and can vary depending on the context in which it is played. By summarizing the main factors that affect EHS and the methodologies used in this field of study, the present review of the literature highlights the fact that a) to ensure effective sight reading, the EHS must be adaptable and optimized in size (neither too long not too short), with the best sight readers exhibiting a high level of perceptual flexibility in adapting their span to the complexity of the score; b) it is important to interpret EHS in the light of the specificities of the score, given that it varies so much both within and between scores; and c) the flexibility of EHS can be a good indicator of the perceptual and cognitive capacities of musicians, showing that a musician's gaze can be attracted early by a complexity in a still distant part of the score. These various points are discussed in the light of the literature on music-reading expertise. Promising avenues of research using the eye tracking method are proposed in order to further our knowledge of the construction of an expertise that requires multisensory integration.

## Introduction

In sight-reading tasks, musicians have to play a score at first sight or after very little preparation (78). This requires them to coordinating visual, auditory and motor processing in order to convert a visual code into a series of motor responses as the score is played ( 69). This activity is highly demanding in terms of perceptual and memory resources. Therefore, fluent sight reading requires musicians to adopt strategies that enable them to optimize their information gathering and performance execution. For example, to facilitate the performance of sequences of notes, instrumentalists use the fingering that is most suitable to perform the score that is to be played (68). In the same way that suitable fingering facilitates the production of notes, is visual information gathering facilitated by a particular eye position during sight reading?

Since the pioneering research by Jacobsen (31) and Weaver (75) on eye-movement behavior during music reading, a growing number of studies have attempted to characterize what constitutes an optimal visual strategy for information processing during music reading (16; 20; 64). Indeed, in a visual task, eye movements are thought to reflect the engaged attention and cognitive processing (for eye-mind theory, see 32; 60), even if some fixations are not “cognitive” in the sense they do not convey any semantic information, but rather perceptual information used for visual guidance. These movements consist of fixations (pauses in the eye movements during which the visual information is processed) and saccades (movements of the eyes during which no or very little visual information is processed; 27). Sight reading therefore requires discontinuous eye behavior (fixations) within a continuous musical timestream (the performance). Furthermore, in cases where the tempo with which a score is played increases, the precision with which the notes are played can be impaired (17). In fact, there is a conflict between, on the one hand, a defined, limited musical timestream and, on the other, the variable time required in order to decode a note.

The challenge facing the musician is to have adequate perceptual and memory resources between reading and playing each note in order to prepare a motor response which respects the constraints of the score ( 33). The eye is thus rarely positioned on the note which is currently being played but instead tends to be further ahead (on the note before it is played; 72). The offset corresponding to the distance or latency between the eye fixation on the score and the musical production by the musician is termed the "eye-hand span" (EHS; 22; 23; 72). The EHS is a measure consisting of multimodal components obtained by synchronizing the eye movements and the musical performance. It makes it possible to observe the relation between what musicians see and what they play and to infer the strategies they adopt. Thanks to advances in the eye-tracking techniques, half of the studies addressing EHS in music reading have been published over the course of the last ten years (see the section “Article selection”) and emphasize the multifactorial nature of EHS. These studies have assessed the trend of EHS to vary as a function of the musician's skill level, the difficulty of the score and the context in which the musical task is performed. Furthermore, a wide range of methodologies have been used in studies of music reading ( 52), both in terms of the definition of the level of expertise of the groups and in the type of musical material chosen. This is particularly the case of studies relating to EHS. The aim of this review is to provide a methodological and theoretical summary of the measurement of EHS and its role in the evaluation of music reading.

## Article selection

The articles included in this review had to 1) contain a music-reading task with a measure of EHS, 2) be published in English, and 3) have undergone peer review. The limited number of articles in this field explains why we decided to include all the studies conducted on music reading and EHS (and not only those conducted using eye-movement recordings) without any restriction in terms of year of publication. This review is based on studies published up to and including June 2021. The following groups of keywords were used in the relevant database Web of Sciences: "eye hand span" and "music". Of the 15 studies in this review (see Table 1), 13 were conducted on pianists (1Adachi et al., 2012; 7; 12; 22; 23; 28; 42; 51; 62; 66; 67; 72; Weaver, 1943), one of which also administered a sight-reading task to a population of singers (12). One study was conducted on violinists (80) and one on xylophonists (44). The two studies by Sloboda (66, 67) were conducted without using eye-movement recording technique.

**Table 1:** Selected papers: peer-reviewed scientific journal articles on eye-hand span in music reading published in English since 1943

Author(s)	Year	N	Journal	Title
Weaver	1943	15	*Psychological Monographs*	Studies of ocular behavior in music reading
Sloboda	1974	10	*Psychology of Music*	The eye-hand span—an approach to the study of sight reading
Sloboda	1977	6	*British Journal of Psychology*	Phrase units as determinants of visual processing in music reading
Truitt, Clifton, Pollatsek, & Rayner	1997	8	*Visual Cognition*	The perceptual span and the eye-hand span in sight reading music
Furneaux & Land	1999	8	*Proceedings of the Royal Society of London*	The effects of skill on the eye-hand span during musical sight-reading
Gilman & Underwood	2003	40	*Visual Cognition*	Restricting the field of view to investigate the perceptual spans of pianists
Wurtz, Mueri, & Wiesendanger	2009	7	*Experimental Brain Research*	Sight-reading of violinists: Eye movements anticipate the musical flow
Adachi, Takiuchi, & Shoda	2012	18	*12th international Conference on Music Perception and Cognition Conference Thessaloniki, Greece*	Effects of melodic structure and meter on the sight-reading performances of beginners and advanced pianists
Penttinen, Huovinen, & Ylitalo	2015	38	*International Journal of Music Education: Research*	Reading ahead: Adult music students’ eye movements in temporally controlled performances of a children’s song
Rosemann, Altenmüller, & Fahle	2016	9	*Psychology of Music*	The art of sight-reading: Influence of practice, playing tempo, complexity, and cognitive skills on the eye-hand span in pianists
Cara	2018	22	*Musicae Scientiae*	Anticipation awareness and visual monitoring in reading contemporary music
Huovinen, Ylitalo, & Puurtinen	2018	37	*Journal of Eye Movement Research*	Early attraction in temporally controlled sight reading of music
Marandola	2019	30	*Journal of Eye Movement Research*	Eye-hand synchronisation in xylophone performance: Two case-studies with African and Western percussionists
Lim, Park, Rhyu, Chung, Kim, & Yi	2019	31	*Scientific Reports*	Eye-hand span is not an indicator of but a strategy for proficient sight-reading in piano performance
Chitalkina, Puurtinen, Gruber, & Bednarik	2021	24	*International Journal of Music Education*	Handling of incongruences in music notation during singing or playing

## Origins of the EHS measure

EHS is not a measure specific to music; it has also been used to measure the performance of typists (6) and was inspired by the eye-voice span, which corresponds to the number of words separating the ocular activity from the word during text reading (5; 30; 38; 53) or singing (12; 25). Weaver (75 was the first author to adapt EHS to music reading by using a photographic method which made it possible to record the position of the eyes on a score that was to be played at the piano. He was able to observe that the number of notes (from 1.9 to 3.1 on average) and chords (1.5 on average) between the eye fixation on the note and its motor production was not always the same for all musicians. Other studies (66; 67) conducted some decades later attempted to identify the causes of this variability by applying to music reading a method used by Levin and Kaplan (40) to measure the eye-voice span in text reading. This method consisted in presenting a text to be read aloud and switching off the light source that was illuminating the text during reading. The reader then recited all the words in the text that he or she had been able to perceive before the light was switched off. Thus, the number of correctly spoken words after the last word produced at the moment the light was switched off corresponded to the eye-voice span (between 4 and 5 words in this study). In studies of music reading (66; 67), EHS corresponded to the number of correctly played notes after the score had been "switched-off". This method for measuring EHS can be criticized (23; 72) for not taking account of the ability of musicians to benefit from a priming effect or make inferences concerning the continuation of the played melody. It consequently probably overestimates the span (73). It is difficult to distinguish musicians' EHS from their ability to infer the music without using an eye-movement tracking method. All the studies of EHS conducted since that of Sloboda (67) have used the eye-movement recording method.

## Why is EHS a suitable measure for evaluating sight reading?

To evaluate the behavior of musicians in a music-reading task and describe the oculomotor strategies which lead to good performance, it is necessary to use fine-grained methods which are able to predict the musicians' level of expertise and the effects related to the specific characteristics of the task (e.g., complexity, context). These measures can then be used to distinguish between the musicians as a function of their skills and between the scores as a function of their complexity. Among the eye movement variables used to assess music reading behavior, EHS and perceptual span are examined in order to evaluate the perceptual capabilities of musicians (23; 43; 59; 72; 79). In a visual task, the perceptual span represents the quantity of information perceived in the region of the visual field around the fixation point (foveal and parafoveal regions) and within which the useful information is extracted (59; 64), whereas EHS corresponds to the distance between the musician's perception and production (43). While the perceptual span can be applied to any task that involves the visual modality, EHS has a multimodal dimension and can be used in tasks involving typing (6), video games (48) or sight reading (72).

### Perceptual span: measure of perceptual capabilities in a visual task

There are many fields in which perceptual span is used to measure perceptual capabilities (e.g., radiology, chess, reading). Most studies have shown that individuals' perceptual span depends on their perceptual capabilities specific to the type of task they are performing, which are generally acquired through years of practice (36; 61; 64). Thus, experts are able to encode larger quantities of domain-specific visual patterns called chunks (processing groups of elements that have a strong mutual association as a single unit; 9; 10, 11; 37). Experts therefore generally possess a larger span (for a review, see 61). Measuring the perceptual span in a reading task is not as simple as it is in other visual tasks. Indeed, the attentional focus is continuously moving over the upcoming words (text reading) or notes (music reading) ( 54). The eye-contingent moving window technique developed by McConkie and Rayner (45) can be used to measure the perceptual span (for a review, see 55). This paradigm consists in reducing the visual presentation of a text to be read by leaving visible only a window of a few characters to the right of the fixation position. When the eyes move over the text, the window of visibility moves towards the characters that are being looked at. By varying the size of this window, it is possible to determine the threshold value for the number of characters as of which any window of smaller size will render reading less fluent and at which any larger window will result in the same reading capacity as when visibility is total. Thus, perceptual span corresponds to the minimum number of characters that readers need if they are not to be disturbed in their reading. This paradigm has made it possible to observe that in the case of text reading, the span increases with mastery of language (13) or diminishes in the presence of unforeseen or complex elements ( 19). Perceptual span is therefore a discriminant measure which is sensitive both to an individual's capabilities in a visual task and to the characteristics that cause the complexity of the task to vary.

### EHS: a complementary measure of perceptual span during sight reading

As in the case of text reading, perceptual span also increases with music-reading expertise (4; 73). In a study by Waters et al. (73), the notes contained in chords presented briefly in the visual modality were recalled better by the most skilled musicians. These results confirm that perceptual span is a measure that is dependent on the specific knowledge present in memory, including in music-reading tasks. However, music reading may involve various tasks which differ in their cognitive demands (52). In the experiments conducted by Waters et al. (73) and Burman and Booth (42009), musicians performed a note detection task unaccompanied by any associated musical performance. Now, whether reading is associated with musical production or whether it is silent, or whether performance is produced at first sight or once the score is known, these variations in cognitive demand impact visual processing and the perceptual span measure can also be impacted. Indeed, even though a silent music-reading task can involve sensorimotor processing that is similar in some points to a music-reading task associated with motor production (70), sight-reading tasks are specific in that the musicians’ attention continually shifts to the score as they decipher it and they involve motor activity resulting in actual sound production (65).

More specifically, Truitt et al. (72) and Gilman and Underwood ( 23) measured perceptual span using an adaptation of the gaze-contingent moving window method. Pianists performed a sight-reading task. During this task, the size of the window over the score was varied in order to measure the perceptual span. The results showed that the musicians' perceptual span was greatly reduced (from 2 to 4 beats to the right of eye fixation) and did not vary as a function of expertise ( 23; 72) or task complexity ( 23). The absence of any effects of expertise and complexity on perceptual span may appear surprising given the robust nature of these effects in other activities (i.e., text reading, playing chess). There are various ways of interpreting these results. First of all, Truitt et al. (72) refute the hypothesis of a threshold effect linked to the relatively simple nature of the material (scores of a single staff) since the less skilled pianists exhibited poorer performances than the skilled and themselves reported that they did not find the employed musical material to be particularly easy. Furthermore, in these two studies, the authors revealed effects of complexity (23) and expertise (23; 72) on EHS, indicating that the skilled musicians did indeed enjoy a perceptual advantage over the less skilled (see sections "The effect of expertise" and "Complexity"). Furthermore, Gilman and Underwood (23), who used more complex material, interpreted the absence of expertise and complexity effects on perceptual span in the light of two hypotheses: Either a sight-reading task imposes such rhythmic and temporal constraints that it is necessary for musicians to reduce their perceptual span in order to avoid overloading working memory during the task, or the skilled musicians do indeed have a larger perceptual span than the less skilled, but this difference is not observed in the quantity of visual information contained in this span during sight reading but instead in the type of information contained in the perceptual span. For example, skilled musicians might perceive information associated both with the pitch and rhythm of a note, whereas less skilled musicians would perceive only one or other of these types of information. Measuring perceptual span using the eye-contingent moving window method therefore comes up against its limits in a sight-reading task due to its inability to discriminate behaviors as a function of the skills required by and characteristics of the music-reading task (this is not to say that perceptual span does not vary with sight-reading expertise and complexity, but that the eye-contingent moving window method does not appear to discriminate between musicians on the basis of their musical skills and scores on the basis of their complexity), whereas the EHS measure does indeed seem to be discriminant.

Moreover, EHS is a coherent measure given that in a sight-reading task, the conversion of visual symbols into motor production makes it necessary to keep both visual and motor information active in working memory (22; 34, 35). Thus, during sight-reading, the musician's choice of eye position on the score is subject to a dilemma. The eye must be sufficiently far ahead compared to the execution of what currently has to be played in order to anticipate and plan the performance of the score. However, the eye must not be too far advanced from current production in order to avoid creating a mental overload (22; 59). That is why by taking account of the motor modality, the measurement of EHS makes it possible to contextualize the observation of eye movements in the light of what is being played and to infer from this the strategies chosen by the musician note by note.

**Figure 1.**
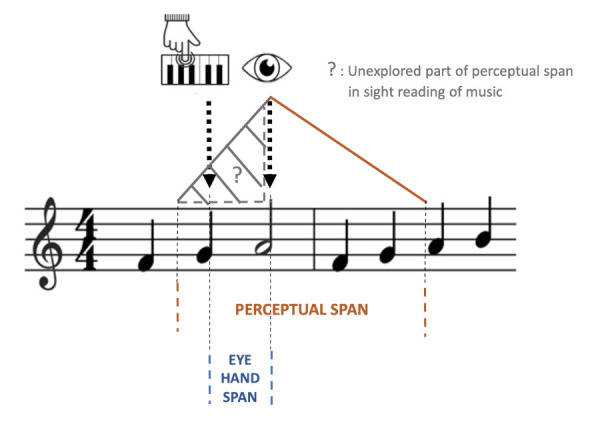
EHS and perceptual span during a sight-reading task

In general, EHS is a measure that is being used more and more to assess sight-reading performances, both because it is sensitive to musicians' skill levels and because it is compatible with the sensory modalities involved in the sight-reading process. EHS is in some way a perceptual span with multimodal components which evaluates sight reading by taking account of the quantity of visuo-motor information to be manipulated. Furthermore, some authors (23; 72) have established the limits of the eye-contingent moving window method. This makes it possible to evaluate the quantity of information present in the perceptual span to the right of the fixation position without providing any information about the extent of this span to the left of the fixation position (see *Figure 1*). These authors emphasize the fact that perceptual span and EHS can complement one another: by combining EHS and perceptual span, it is possible to determine the quantity of information that musicians are able to use during sight reading (see Table 2).

## How to measure EHS?

Two types of EHS measure have been used in the various studies: 1) measures of distance which make it possible to evaluate the quantity of information manipulated by musicians between the moment when they fixate a note and the moment they play it. These measures are obtained by measuring the distance between the fixation point and the virtual position of the hand on the score; and 2) latency measures used to evaluate the time during which the information is maintained in working memory. These are obtained by measuring the latency between the moment when a note is fixated and the moment when it is played. Furthermore, these two measures – of distance and latency – can be divided into two subtypes: measures made in absolute units and measures made in musical units (see Table 2).

### Distance in absolute space

**Figure 2.**
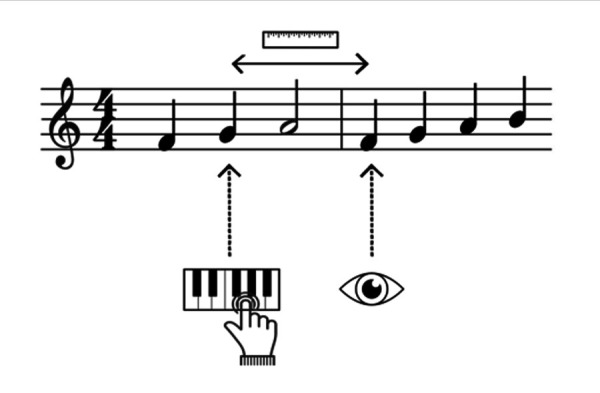
Representation of EHS measured in absolute space

The distance measurements of EHS in absolute space consider the distance between the location of the note which is currently being played and the location of the musician's eye fixation at a given moment ( 72; see *Figure 2*). If a note is played during a saccade, the location of the fixation which follow this saccade are taken into account (23). This measure is expressed in pixels (23; 72) or in mm (23). Very few studies have used the distance in absolute space to measure EHS (see Table 2) because it varies as a function of the size of presentation of the visual score and is therefore not very relevant in itself. That is why, whenever it is used, authors convert it into musical units.

**Table 2:** Eye-hand span measurement method by study

STUDIES	LATENCY		DISTANCE				
	ABSOLUTE	MUSICAL UNITS	ABSOLUTE		MUSICAL UNITS		
	MS	BEATS	PIXELS	MM	NOTES	BEATS	EVENTS
Weaver, 1943					X		X
Sloboda, 1974					X		
Sloboda, 1977					X		
Truitt et al., 1997			X			X	
Furneaux & Land, 1999	X				X		
Gilman Underwood, 2003				X		X	
Wurtz et al., 2009	X				X		
Adachi et al., 2012					X		
Penttinen et al., 2015	X	X					
Rosemann et al., 2016	X					X	
Cara, 2018					X	X	
Huovinen et al., 2018	X	X					
Marandola, 2019					X		
Lim et al., 2019	X				X	X	
Chitalkina et al., 2021	X						

### Distance in musical units

**Figure 3.**
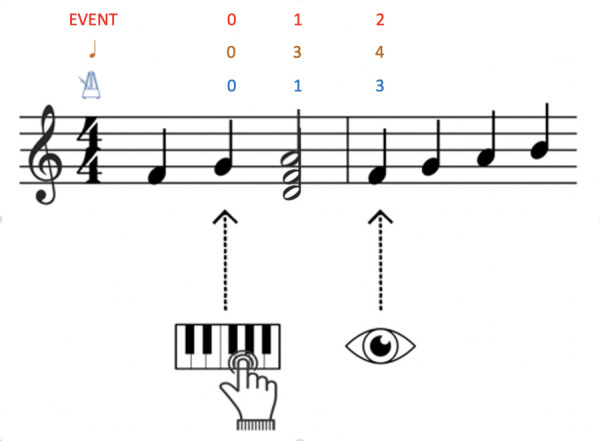
Representation of EHS measured in musical units. In red the EHS measured in number of events, in orange the EHS measured in number of notes, and in blue the EHS measured in number of beats.

These measures evaluate the distance in musical units between the note that is currently being played and the note which is being fixated by the musician at a given moment (see *Figure 3*). EHS in musical units is expressed either in notes (1; 7; 22; 42; 66; 67; 75; 80), in beats (7; 23; 42; 62; 72), or in events (i.e., notes, chords or pauses read in the score; 75). This measure is obtained by freezing the musical time course. For each played note, this defines the distance of the eye on the score. Huovinen et al. (28) named it a "forward projective approach" because this measure is performed in the direction of reading and is time-locked to action: at any given time, the played note is the initial measure, and the aim is to measure how far ahead the eye is from this initial measure. Furthermore, depending on whether the span is measured in notes, beats or events, this measure is sensitive to different aspects of the score. The measure made in terms of notes considers each note to be a single unit of the span independently of its temporal value (in *Figure 3*, the Dm chord represents three span units because it consists of three notes) whereas the measure in beats considers each beat to be a single span unit independently of the number of notes per beat (in *Figure 3*, the Dm chord represents two span units because it lasts for two beats). Finally, the measure in terms of events considers all the events that occur simultaneously to constitute a single span unit independently of their temporal value and number (in *Figure 3*, the Dm chord represents a single span unit because it consists of notes which occur simultaneously). Thus, one and the same observation can give rise to different values of EHS (here, four notes, three beats or two events). We assume that it is preferable to choose the method used to measure EHS in the light of the material used for the experiment. When the written music consists to a large extent of chords formed from different numbers of notes, EHS in beats should be used, whereas when there are primarily notes on their own, EHS in notes would be preferable. EHS in events can be used in both the above cases but comes up against its limits when there is considerable variation in the temporal value of the different events. Even though these measures are different, there are experimental conditions in which they are equivalent, for example in situations in which the score consists solely of quarter- notes without chords, the value of EHS in notes, beats or events is the same.

### Latency in absolute time

**Figure 4.**
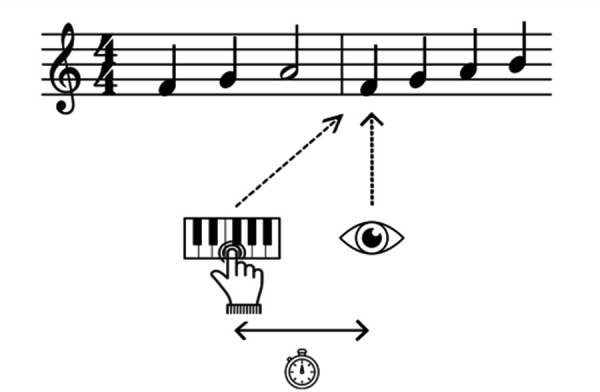
Representation of EHS measured in absolute time

The measure of EHS latency in absolute time considers the time that elapses between the fixation of a note and its execution on the instrument (see *Figure 4*). This measure is expressed in milliseconds (ms) (12; 22; 28; 42; 51; 62; 80). Unlike the measures of distance, measuring the latency in absolute time is considered to be a "single-item lag approach" in so far as it corresponds to the time difference between the reading and playing of the same note ( 28). Initially proposed by Furneaux and Land (22), this measure is complementary to those which measure distance and is used to evaluate the time necessary to decode the note and keep it active in memory.

### Latency in musical units (Eye-Time Span)

**Figure 5.**
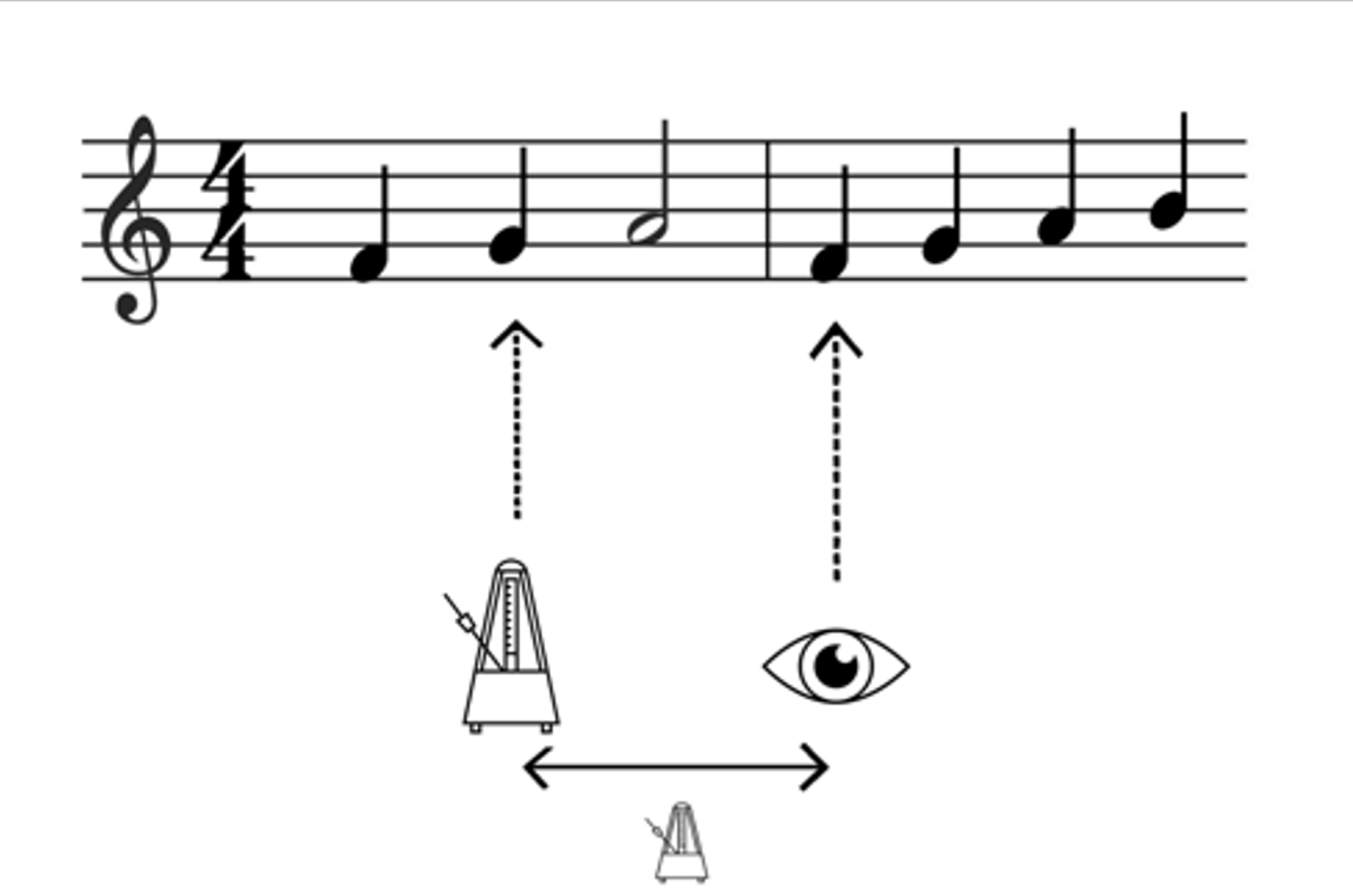
Representation of EHS measured in musical units

Measuring latency in musical units (ETS) is a specific form of measure in that it does not take account of the musician's musical performance and it requires the use of a metronome during the experiment. This measure corresponds to the difference between the temporal occurrence of the fixated note and the virtual position of the metronome on the score and is expressed in beats (28; 51). To measure this value, each note is associated with a temporal occurrence which is determined by the tempo at which the score has to be sight-read. Thus, when the eye fixates a note which corresponds to the fifth beat in the score at the same time as the metronome is only at the second beat since the start of the score, the value of the ETS is three beats (see *Figure 5*). Here, the ETS constitutes a so-called "backward projective approach" because the measure is performed in the direction opposite to reading. The starting point for each measurement is a fixation on a given note, and the aim is to measure how far it is from the virtual position of the metronome. The ETS seems at first sight to be similar to the EHS measured as a function of the distance in musical units when the musician respects the score's tempo and rhythm. Although the two approaches yield very similar results, the interpretation of span must change depending on the way it is measured. The ETS differs from usual EHS measurements in that the first is time-locked to fixation and the latter are time-locked to action (key presses). Thus, EHS might be imprecise, because the fixation typically occurs slightly earlier or later than the execution of the note (28). The ETS is of value for measuring local changes in musicians' eye-movement patterns since it evaluates their tendency to distance their gaze from the virtual position of the metronome on the score, for example in order to manage the occurrence of a difficulty (see section "Effect of a temporary complexity on EHS: the attraction hypothesis").

## The main factors involved in eye-hand span variability

Initially, EHS was used to answer a simple question: what is the distance between the note being played and the fixation point on the score? (75). The answers to this question differ depending on the factors manipulated and the way they are defined. Indeed, the distance by which the eye precedes the hand involves multiple factors. It is not always relevant to define a precise value for EHS in musicians but instead to identify each of the factors that can explain this variability in order to be able to interpret it. It is possible to identify three main types of factors which have an effect on EHS: those which depend on the musician's expertise level, those which depend on the complexity of the score and those which depend on the context in which the task is performed (see *Figure 8*).

## Factors which depend on the musician's expertise level

There is a wide range of musician’s dependent factors that has been studied to interpret sight-reading skills. Both general cognitive performance and domain-related skills have shown to explain sight-reading efficiency. On the one hand, positive correlations between sight-reading performances and working memory capacities (39; 46), processing speed (34; 39; 46) and pattern recognition (74) have been observed. Since EHS is a measure of the management of multimodal information, it seems reasonable to assume that general cognitive performance is to some extent correlated with this indicator. Musicians with greater working memory capacities would be able to maintain more notes active in memory and would therefore have a larger EHS than musicians with poorer working memory capacities. On the two studies that have measured these two variables, Cara (7) has shown that the musicians with the largest EHS also exhibited better performances in terms of visuo-spatial working memory (Corsi block-tapping test), whereas the results of Rosemann et al. (62) did not support this hypothesis. It is therefore quite difficult to conclude on this aspect of EHS variability, but it would be interesting that more studies investigate the way general cognitive performances impact EHS. On the other hand, music reading expertise is one of the main factors that has been studied to explain sight reading efficiency. In fact, studying the behavior of skilled musicians and, more particularly, their eye movements makes it possible to provide information about the task-performance strategies they adopt (14; 26). Part of the aim of studying EHS as a function of expertise is to understand how musicians' eye movements should adapt in order to ensure the effective decoding of a score. The theories of expert memory postulate that expertise in a domain structures perception. Skilled musicians would preferentially encode information by means of a chunking mechanism (10, 11) as well as by establishing a relation between the information to be processed and knowledge schemas previously integrated in long-term memory and acquired over the course of hours of learning (15; 18; 24; 77). In music reading, the expert ability to process information more in the form of chunks than as individual events (74) makes it possible to hypothesize that EHS will be greater among skilled than less skilled musicians.

### Differences in the definition of expertise: Learning vs. playing level

In all, nine studies have measured the effects of expertise on EHS. We noted that expertise in sight reading has been defined in ways that differ depending on the EHS study (see *Figure 8*). These methodological differences may be a source of variability in the measurement of EHS and therefore, it seems important to us to start by mentioning them. Among the nine studies which have examined the effect of expertise on EHS (see Table 3), we have identified two main types of expertise definition: definitions based on the length of time that musicians have spent learning their instrument, which we shall refer to as "learning level" (four studies) and those based on the quality of the musician's performance during the music-reading task performed during the study, which we shall refer to as "playing level" (five studies). Sloboda (66) was the first author to measure the effect of expertise on EHS. With expertise being defined as a function of "playing level", the participants who made the fewest errors during the sight-reading task were considered to be the most skilled. Generally speaking, the studies which distinguish between skilled and less skilled musicians as a function of playing level count either, like Sloboda (66), the number of errors made while playing (number of wrong, omitted, invented notes; 7; 23; 42), or the mean execution time for a metrical division (a bar, a phrase; 7; 72): the musicians who take the least time to play a metrical division have been considered to be the most skilled. With regard to the studies which have used the "learning level" criterion to define the level of music-reading expertise, these have taken account of the musician's position within a music academy (28; 51) or simply the number of years spent practicing an instrument ( 1), with the musicians who have spent longest learning their instrument being considered to be the most skilled.

However, depending on the experimental design of the sight-reading studies, some musicians can move from one expertise group to another. For example, some studies have selected participants who are all at a similar level in their music studies and who are then divided into two groups depending on their playing proficiency. This is the case in a study by Lim et al. (42) among musicians who had received more than ten years of training. These musicians were distributed into two groups based on their performance in a sight-reading task ("playing level"). However, the musicians who were considered to be less skilled in this study would have been placed in the skilled category in the study undertaken by Adachi et al. (1), who took account of the number of years of music study in order to define expertise ("learning level"). Similarly, in a task involving the sight reading of contemporary music (7), some students, categorized on the basis of the playing-level criterion, formed part of the skilled group and some professionals were assigned to the less skilled group.

Both the "playing-level" and the "learning-level" criteria are legitimate ways of defining music-reading expertise. However, it seems important to point out that these differences in definition can be problematic from the methodological point of view since they might lead to different observations depending on the way expertise is determined. Combining the playing- and learning-level criteria might be a good way to reveal the processes and strategies that make good sight reading possible. Whichever way the level of expertise is established, it is crucial to take account of it when interpreting and discussing the results of the study.

### The effect of expertise

Despite the differences in the ways of categorizing the groups of expertise, there is a consensus on the fact that EHS (distance) is greater on average among skilled than less skilled players (Adachi et al., 2012; 7; 22; 23; 28; 42; 51; 62; 66; 72). It is, nevertheless, difficult to define a precise EHS as a function of expertise given that it varies so much depending on the way it is measured in the various studies (see Table 3). EHS measured in notes ranges from 0.52 to 3.69 notes for novices (1; 7) and from 1.73 to 6.8 notes for skilled musicians (1; 66;), whereas that measured in beats ranges from 0.75 to 2.10 beats for novices (7; 23) and from 1 to 2.85 beats for skilled musicians (7; 23). However, it is necessary to point out that these differences all operate in the same direction: in all cases, the values are higher for skilled than less skilled musicians.

Nevertheless, even if the distance by which the eye is ahead of the hand is greater in skilled than less skilled musicians, the latency between the moment when musicians fixate a note and the moment when they play that note does not necessarily seem to vary as a function of expertise. In a study conducted by Furneaux and Land (22), musicians of different levels of expertise had to sight-read scores. The results showed that expertise impacted EHS (distance) but not EHS (latency). It therefore seems that the latency between the played and read note is similar in skilled musicians and novices and that the difference is to be found in the quantity of information processed in advance, with skilled musicians being able to process more information in their EHS. Given that the tempo was imposed in this study, the fact that the skilled musicians have a larger EHS (distance) than the less skilled while the EHS (latency) was the same for both expertise levels is a little bit counterintuitive. These results might be explained by differences in speed of execution between skilled and less skilled musicians even though tempo was imposed. When the speed of execution increases, more notes are contained in one time-step. Thus, musicians who do not play at the same tempo may have EHS that differ in terms of distance but have an identical latency (see the section on the effect of tempo on EHS). Penttinen et al. (51) obtained result

**Table 3:** Eye-Hand Span as a function of expertise level

STUDIES	SUBJECTS			EXPERTISE CRITERION	LATENCY		DISTANCE			
	LS	I	S		ABSOLUTE	MUSICAL UNITS	ABSOLUTE		MUSICAL UNITS	
					MS	BEATS	PIXELS	MM	NOTES	BEATS
Sloboda, 1974			10	Playing Level (LS-S)					3.6 – 6.8 **	
Truitt et al., 1997	4		4	Playing Level (LS-S)			11 – 42 **			1 – 2 **
Furneaux & Land, 1999	3	3	2	Learning Level (LS: g.3/4 - I: g.6/7 - S: acc.)	1000 NS				2 – 2.5 – 3.75 **	
Gilman & Underwood, 2003	13		17	Playing Level (LS-S)				15 – 19 *		0.75 – 1 *
Adachi et al., 2012	9		9	Learning Level (LS: 9.22 yop - S: 16.22 yop)					0.52 – 1.73 ***	
Penttinen et al., 2015	14		24	Learning Level (LS: 11.5 yop S: 14.8 yop)		(0-1-1+ Beats) LS 36 – 56.1 – 7.8 % S 30 – 55.7 – 14.3 % ***				
Cara, 2018	11		11	Playing Level (LS-S)					3.69 – 4.70 *	2.10 – 2.85 *
Huovinen et al., 2018	14		23	Learning Level (LS: 11.3 yop S: 14.8 yop)		S = LS + 0.29 to 0.53 ***				
Lim et al., 2019	10	11	10	Playing Level	r = .26 **				NS	r = .22 *

LS: Less Skilled; I: Intermediate; S: Skilled; yop: years of practice; g.: grade standard (as used by the Associated Board of the Royal School of Music); acc.: accompanists; r: Spearman Coefficient; NS: Non-Significant *: *p* < .05; **: *p* < .01; ***: *p* < .001

similar to those of Furneaux and Land (22). However, the observed EHS (latency in metrical units) was variable for any given subject and any given score. Indeed, for 30% of the sight-reading session, a "zero-span" was observed (irrespective of their level of expertise, the musicians fixated the note that they were currently playing), whereas for 56% of the session, the musicians fixated one beat ahead of what they were currently playing. Furthermore, the skilled musicians used an "extended span" (more than one beat) for 14.3% of the time, as compared with 7.8% for the less skilled players (see Table 3). The authors suggest that while the differences in temporal EHS as a function of expertise are not observed on average across the score, an analysis of local EHS could make it possible to differentiate between behaviors, with the skilled musicians exhibiting more extended spans.

Moreover, Lim et al. (42) studied the change in musicians' eye-movement strategies as a function of their expertise during more or less complex scores. They succeeded in observing an ability to adapt EHS as a function of expertise during the reading of scores of two levels of complexity: the size of the EHS of the musicians with the best sight-reading performances was negatively correlated with score complexity. The EHS of those with the best performances increased when the score was easier and fell when it was more complex, a pattern which was not observed in the lower-performing participants. The results indicate that if EHS is to be optimum, it must be possible to modulate it in the light of the score to be played, and in particular in the light of its complexity. Thus, musicians with good sight-reading skills seem to benefit from perceptual advantages that make it possible to adapt their EHS across the score in order to avoid mental overload ( 22; 42).

Expertise is therefore a determining factor in the development of EHS. Nevertheless, it seems justifiable not to consider expertise to be the only factor contributing to variation in the span and to complement the measurement of expertise with a consideration of the specific characteristics of the score.

## Factors which depend on the score

### Differences in the employed musical material

It is necessary to point out that the various studies that have measured EHS in pianists have not always used the same type of musical material. Some authors have preferred to use somewhat simple material: a melodic line from a piano score played with the right hand and consisting only of diatonic notes (51; 67; 72), sometimes with most of the notes being diatonic neighbors of the preceding ones (step-wise; 12; 28). At the methodological level, this type of score is not representative of the scores that skilled musicians may encounter during their everyday activity. Nevertheless, this approach has the advantage of simplifying the measurement of EHS. Firstly, the notes in these scores are not all concentrated together as they may be in more complex scores with notes being stacked in chords or having a variety of time values (cf., measurement bias presented in the section *"Distance in musical units");* secondly, since the reading of a musical score on two staffs is characterized by movements up and down between the top and bottom staff (62; 75), the absence of the bass staff prevents the occurrence of vertical eye movements during EHS measurement. However, current eye-tracking methods are considerably improved compared to those used in the initial studies involving EHS measurements and thus make it possible to target the areas of interest (AOIs) more accurately. There can be no doubt that even though a piece played with one hand makes it possible to control for potential motor complications in the execution of the tune (which would have a negative impact on the processes involved in the ocular processing of the score; 51), it is not representative of the complexities that musicians must confront during sight reading. Indeed, the choice of hand position and fingering is an integral part of the motor component of a sight-reading task (16; 50). By contrast, other studies have used more ecological scores, which have often been more complex (with violinists, 80) or have been written on two staffs (in pianists, 1; 7; 22; 23; 42; 62; 75). These two methodological approaches both have advantages for the study of EHS. Simple material facilitates the measurement of EHS, whereas more complex material makes it possible to observe the ocular behavior of musicians in more ecological situations that are closer to their real-life activity.

### Complexity

In a sight-reading task, complexity usually brings about an increase in the number of execution errors (41) explained by the increased mental workload (71) induced in the musician. Complexity is the most frequently studied factor in the articles on EHS (eleven studies) and a wide variety of forms of complexity have been studied.

### How is musical complexity defined?

Complexity is not always defined in the same way in the studies which have measured EHS (see Table 4; *Figure 8*). It may depend on low-level perceptual characteristics such as the legibility of the score or on higher-level characteristics such as its musical structure. Some studies have modulated the visual characteristics of the score by physically degrading it, for example by removing physical markers (67;) or by presenting only a reduced visual window onto the score which gradually moves as sight reading proceeds (23; 72; see *Figure 8*). In these cases, complexity depended on the legibility of the score and was not linked to the structural aspects of the written music. Other studies have induced a structural complexity by varying pitch-related and rhythmic characteristics. As far as pitch is concerned, some authors are of the opinion that the number of accidentals (sharps and flats) could work as a criterion of complexity: metrical divisions that contain the largest number of modified notes are thus thought to be the most complex (28; 42). It is also possible to manipulate the type of pattern proposed, with patterns of step-wise notes (e.g., in which most of the notes are diatonic neighbors of the preceding ones) corresponding to non-complex material and patterns of skip-wise notes (e.g., in which a note skips a diatonic step) corresponding to complex material (1; 28). Furthermore, within the set of complexities generated by modifying the pitch of the notes, it is necessary to distinguish between those which respect the rules of tonality (1t al., 2012; 7; 12; 23; 28; 42) and those which violate the musician's musical expectations (12; 51; 67;). Indeed, works on expertise have shown that randomly organized material can cancel out the effects of expertise on the quantity of perceived information (10). One cannot therefore consider complexity to be of the same nature depending on whether or not the score respects the tonal rules of Western music. Furthermore, there are differences regarding the prolonged or temporary nature of the difficulty: some studies have used material in which the complexity resides in a single note (1; 12; 28) or a single metrical division (beat: 51; bar: 51; 62) to compare intra-score EHS, whereas there are others which have used entire complex scores in order to compare EHS between scores ( 1, 7; 23; 42; 67; 72; 80). The prolonged or temporary character of the complexity could therefore be a factor of variability in EHS. Secondly, in order to vary the structural aspects of scores, some studies have modulated the rhythm, modifying the time signature, with less frequent time signatures (5/4) being considered more complex than more frequent ones (4/4 or 3/4; 1t al., 2012). Others have varied the number of notes per metrical division, with divisions containing the greatest number of notes being considered the most complex (7; 42), or the duration of the notes: the more heterogeneous the notes are, the more complex the piece is (80), or, alternatively, the shorter the notes are, the more complex the piece is (eighth-note vs. quarter-note patterns; 51). In their study, Lim et al. (42) proposed an original way of measuring the complexity of a score by measuring the entropy of different pieces as a function of the number of accidentals (pitch), notes and beats per metrical division (rhythm). In information theory (63), entropy is the degree of uncertainty of the values that make up the system. It increases as a function of the possible number of items and the tendency of each item to have an equivalent probability of occurring. In music reading, Lim et al. (42) considered that the possible number of items corresponds to the 12 existing pitch classes. Thus, entropy is greater in a piece when each of the 12 tones appears with equal frequency. Their definition of the complexity of a musical score therefore corresponds to its tendency to be composed of unpredictable notes. In their experiment, simple scores had an entropy of 2,782 bits compared to 3,542 bits in the case of the complex scores.

In the same way that the differences in the definitions of expertise have to be considered when determining the factors which impact EHS, different types and intensities of complexity also have to be taken into account. Generally speaking, and in order to obtain a more fine-grained representation of the effects of complexity on music reading, it would be interesting to elaborate relevant score complexity criteria, while taking account of 1) the various rhythmic and pitch-related aspects which affect a score's structural complexity, 2) the prolonged or temporary nature of the complexity, and 3) the respect or non-respect for tonal rules in the score.

### Effect of prolonged complexity on EHS

Whether induced by a low-level (perceptual; 23; 67; 72) or high-level factor (structural; 23; 80), complexity tends to reduce EHS (see Table 4). Wurtz et al. (80) measured the EHS of violinists performing two scores of different complexities. The results showed that the musicians' EHS was lower for the complex score (three notes) than for the simpler score (six notes). Furthermore, in this same study, the violinists' EHS in absolute time was approximately 1000 ms and did not vary as a function of the complexity of the scores. These results agree with those of Rosemann et al. (62) who also observed no difference in EHS in absolute time as a function of complexity. This seems to indicate that the complexity of the notes influences the distance by which the eyes are ahead of the hand in the score but does not influence the latency between the fixation of the note and its execution (same observations as for the effect of expertise on EHS). Here again, we suppose that the effect of complexity on EHS can be modulated by the tempo. Since complexity tends to reduce the speed of execution (17), a smaller EHS (distance) does not necessarily signify a smaller EHS (latency) since note duration increases as the tempo slows (see section on the effect of tempo on the EHS). The distance by which the eyes are ahead of the hand in the score would therefore constitute the variable part of the measured EHS and depend on the relation between the complexity of a score and the musician's sight-reading abilities.

### Effect of a temporary complexity on EHS: the attraction hypothesis

In music reading as in text reading, one of the main questions relates to the guidance of the eye movements during reading, and in particular when to end gaze fixation and the point to which to move the next eye movement. According to Rayner and McConkie (57), these two parameters depend on different factors based on information perceived in parafoveal regions and are independent of one another. The low-level, non-linguistic visual variables such as word length or inter-word spacing determine where to fixate next, while the difficulty of the words to be processed influences the time at which the eyes are moved. The fixation duration is thus subject to process monitoring guidance ( 58). The time at which the eye has to move away from a word in order to fixate the next word appears to be influenced by the frequency of this word and its predictability as a function of the text that has already been read (high-level factors). The point at which a word is fixated generally appears to depend on the basis of information relating to low-level visual factors (spaces surrounding the word, word length, distance between the launch site and the target word; 56). Hyönä (29) tested the attraction hypothesis, according to which an orthographically infrequent letter (perceptually more salient than a frequent letter sequence) attracts ocular fixation. Thus, an infrequent word ending would attract the eye to a position further on in the word, whereas an infrequent letter sequence at the start of the word would attract the eye to the start of the word (instead of the optimum fixation point, which is generally the center of the word). Consequently, the perceptual complexity of a word in parafoveal vision (e.g., word length, typographic density, frequency of the first trigram) will modify the landing area of the saccade and increase the duration of fixation of the current word since reading makes it necessary to anticipate the processing of the upcoming words. Furthermore, the fixation duration of a word makes it possible to measure the processing time required for this word. This time takes account of oculomotor mechanisms (programming and triggering of saccades) as well as of attentional and psycholinguistic processes. If a visual difficulty is perceived in parafoveal vision, the fixation duration of the current word may increase (30; 76). In music reading, Huovinen et al. (28) tested two hypotheses derived from Hyönä's attraction hypothesis (29). The first supposes that a visually complex note in a score will be fixated earlier ("when") than a note that exhibits no complexity, thus resulting in an increased ETS on the note. This is what the authors referred to as the "Early Attraction Hypothesis". The second hypothesis considers that the eye will be attracted by this difficulty from a further distance away ("where") than in the case of non-complex notes, thereby resulting in an increase in the size of the incoming saccades towards this note. This is what the authors called the "Distant Attraction Hypothesis".

**Figure 6.**
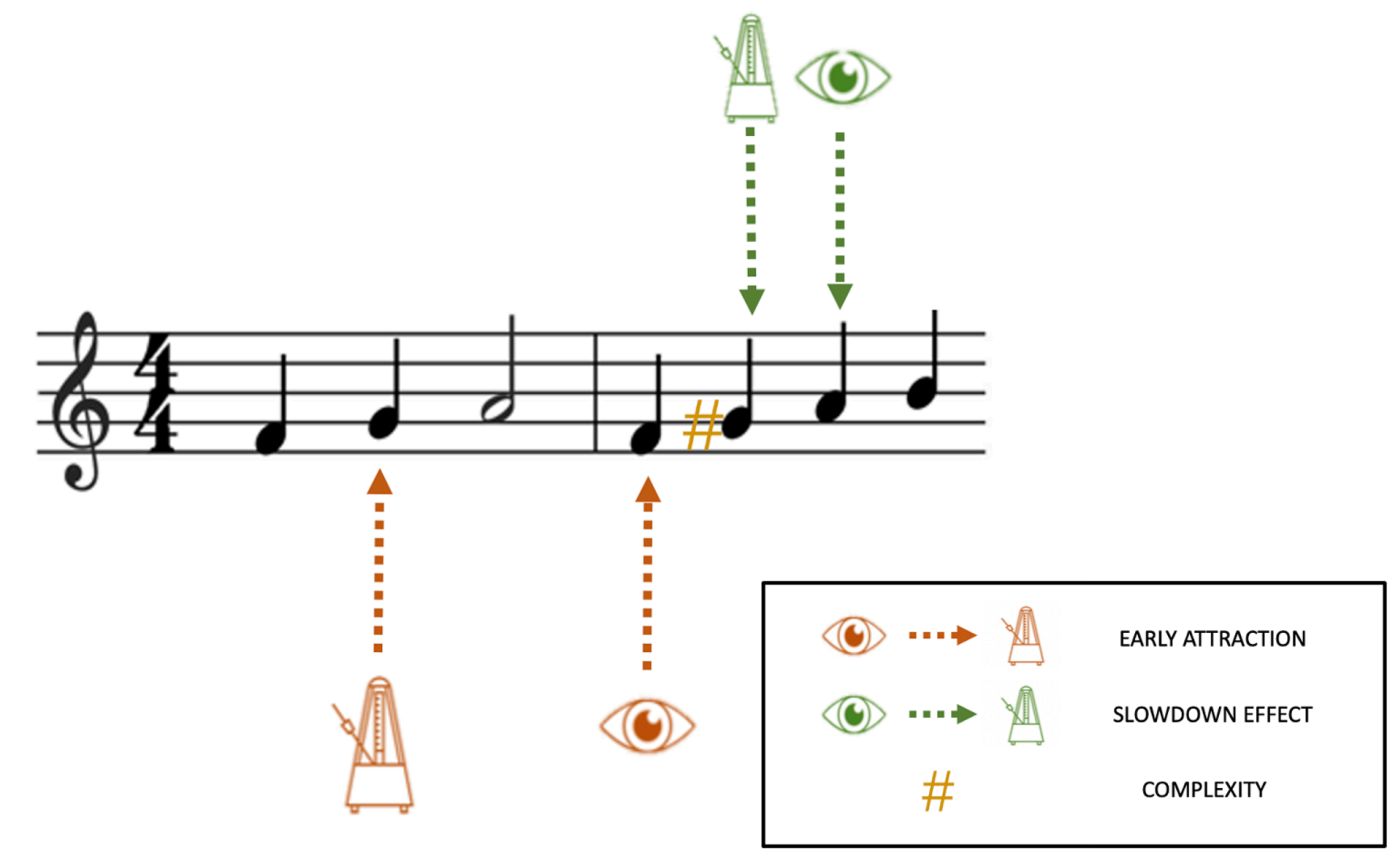
Within-staff variation of ETS as a function of the location of the complexity

To test this hypothesis, they administered sight-reading tasks with an imposed tempo in step-wise and skip-wise conditions. The ETS tended to increase for complex notes. In addition, the size of the incoming saccades towards complex notes was significantly greater than for simple notes. These results tend to argue in favor of the "Early Attraction Hypothesis" and the "Distant Attraction Hypothesis", respectively. In the second experiment conducted by these authors, the same conditions were used but complexity was more salient because the complex note was modified by the presence of an accidental (sharp). In this case, the ETS tended to increase for the notes preceding the skip, indicating that when a difficulty is more salient in the parafoveal region, the ocular adjustment effect can occur for the notes that preceded the difficulty. The distant, early attraction of the eye to the complexity might reflect the need to allow oneself the time necessary to process it. Thus, optimum sight reading depends on the ability to identify upcoming complexities at an early stage. Generally speaking, this study confirmed that the EHS varies throughout one and the same score and that it might be relevant to measure it at the local level in order to observe musicians' adaptive strategies. Furthermore, Chitalkina et al. (12) obtained results similar to those of Huovinen et al. (28) and revealed a reduction in ETS in the region following the complexity, which might indicate that after causing the early attraction of the eye, the processing of the difficulty slows down musicians' eye trajectories compared to the metronome (slowdown effect: see *Figure 6*).

The results obtained in these studies seem to contradict those that have shown a reduced EHS in the presence of prolonged complexities (Adachi et al., 2012; 7; 23; 67; 80). However, they are not incompatible. On the one hand, the method of measurement might affect the way that local complexities impact the EHS. On the other hand, the local increase in ETS for complexity is counterbalanced by the processing time, which slows down the eye trajectory (12). It is therefore possible that a temporary complexity will lead to the observation of a local increase in ETS without this having any impact on its mean value over the score as a whole. We assume that ETS must be interpreted as a function of the type of material used and the prolonged or temporary nature of the complexity (simple score with one complex note vs. score that is complex throughout) in order to gain a fine-grained understanding of changes in eye behavior and the processing strategy used during sight reading.

**Table 4:** Eye-Hand Span as a function of complexity

STUDIES		COMPLEXITY		METHOD	LATENCY		DISTANCE			
					ABSOLUTE	MUSICAL UNITS	ABSOLUTE		MUSICAL UNITS	
					MS	BEATS	PIXELS	MM	NOTES	BEATS
Sloboda, 1977	PERC.	ALTERATION	STAFF	MARKERS (C – LC)					4.9 – 5.1 NS	
Sloboda, 1977	STRU.	PITCH	STAFF	HARMONIC NON-SENSE (C – LC)					4.5 – 5.5 ***	
Truitt et al., 1997	PERC.	REDUCTION	STAFF	WINDOW (2 – 4 – 6 beats – NO MW)			21 - 26 - 30 -29 *			
Gilman & Underwood, 2003 - A	PERC.	REDUCTION	STAFF	WINDOW (1 – 2 – 4 beats – NO MW)				14 - 16 - 17 – 17 **		
Gilman & Underwood, 2003 - B	STRU.	PITCH	STAFF	TRANSPOSITION (C – LC)				12 – 15 ***		
Wurtz et al., 2009	STRU.	RYTHM	STAFF	NOTE DURATION (C – LC)	1000 NS				3.5 – 6 *	
Adachi et al., 2012	STRU.	RYTHM	STAFF	TIME SIGNATURE (5/4 – 4/4)					1.26 – 2.03 *	
Adachi et al., 2012	STRU.	PITCH	NOTE	SKIP-WISE					N/A	
Penttinen et al., 2015	STRU.	PITCH	BAR	STEP DOWN DIVISION		NS				
Penttinen et al., 2015	STRU.	RYTHM	BEAT	NOTE DURATION (C – LC)		C < LC **				
Rosemann et al., 2016	STRU.	RYTHM	BAR	NOTE/DIVISION (C – LC)	1258-1320 NS					0.35 – 0.51 **
Cara, 2018	STRU.	RYTHM + PITCH	STAFF	NOTE/DIVISION + HAND-CROSSING (C – LC)					3.78 – 4.29 ***	2.07 – 2.74 ***
Huovinen et al., 2018 - A	STRU.	PITCH	NOTE	SKIP-WISE – ON TARGET BAR		C > LC *				
Huovinen et al., 2018 - B	STRU.	PITCH	NOTE	ACCIDENTAL – PRE TARGET-BAR		C > LC ***				
Lim et al., 2019	STRU.	RYTHM + PITCH	STAFF	NOTES /DIVISION + ACCIDENTAL (C – LC)	820 -1100 *				NS	1.27 – 1.68 *
Chitalkina et al., 2021	STRU.	PITCH	NOTE	INCONGRUENCY - PRE TARGET-BAR	C > LC ***					
Chitalkina et al., 2021	STRU.	PITCH	NOTE	INCONGRUENCY - ON TARGET-BAR	C < LC *					

C: Complex; LC: Less Complex; PERC: Perceptual; STRU.: Structural; NS: Non-Significant; N/A: Not Available; *: *p* < .05; **: *p* < .01; ***: *p* < .001

## Factors which depend on context of music reading

### Should tempo be controlled for and/or imposed in EHS studies?

**Figure 7.**
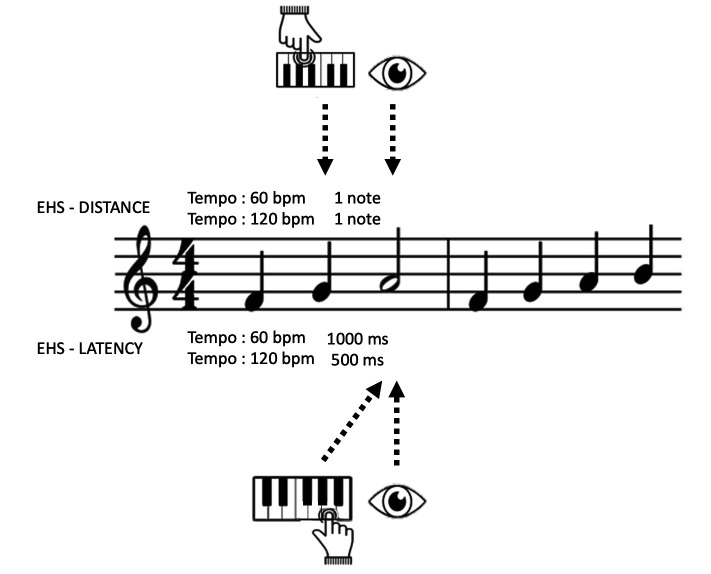
EHS measured in terms of both distance and latency as a function of tempo.

Among the studies that have examined EHS, seven imposed a tempo during the music-reading task (12; 22; 28; 42; 51; 62; 66;) whereas seven others did not ( 1; 7; 23; 67; 72; 75; 80). In the study by Truitt et al. (72), the musicians first had to play the scores with a metronome in order to accustom themselves to a tempo of 152 bpm. In the experimental

part of the task, they had to try to maintain this tempo but without a metronome. Only a few musicians were capable of maintaining the initial tempo showing that when it is not imposed, the chosen tempo differs according to the musician. Furthermore, the time taken to decode a note is a determining factor in sight reading (see introduction) and a "skill/accuracy trade-off" can be observed between the ability to play a score fluently (without making mistakes) and the chosen tempo ( 7; 17). The speed of execution of a score can therefore vary as a function of factors that are specific to the musician (7; 72) or the score (17). However, a note does not have the same duration when played at a slow or fast tempo. Thus, an EHS (distance) equal to a note is equivalent to a longer EHS (latency) in a slow tempo than in a fast tempo (see *Figure 7*). Similarly, the effects of expertise and complexity on EHS in terms of distance and latency could be due to the differences in speed of execution of the score, with skilled musicians looking further on in the score but playing faster and with complexity reducing the distance between the eye and the hand but making it necessary to play more slowly.

We observe two main results among the studies which have measured the effect of tempo on EHS. Firstly, when EHS is measured in terms of musical units, it seems to increase the faster the tempo is, both when it is measured as distance (62) and as latency ( 28). These observations indicate that when the tempo increases, musicians need to direct their gaze further on in the score in order to anticipate more notes and plan their motor actions (see Table 5.

**Table 5:** Eye-Hand Span as a function of Tempo

STUDIES	TEMPO	LATENCY		DISTANCE			
		ABOLUTE	MUSICAL UNITS	ABSOLUTE		MUSICAL UNITS	
		MS	BEATS	PIXELS	MM	NOTES	BEATS
Furneaux & Land, 1999	DEPENDING ON THE STAFF FAST / SLOW	700 / 1300 **					
Rosemann et al., 2016	ORIGINAL DEPENDING ON THE STAFF FAST = O +20% SLOW = O -20%	O = 1342 F = 1143 S= 1475 *				O = 0.42 F = 0.47 S = 0.29 *	
Huovinen et al., 2018	60 VS. 100		F = S + 0.21 to 0.41 ***				
Lim et al., 2019	80 VS 104	NS				NS	

F: Fast Tempo; S: Slow Tempo; O: Original Tempo; NS: Not Significant; *: *p* < .05; **: *p* < .01; ***: *p* < .001

Secondly, when EHS is measured in absolute time, it seems to fall the faster the tempo is (22;62). These observations are consistent with the discussions above (see *Figure 7*) and indicate that despite the fact that musicians fixate a point further on in the score as the tempo becomes faster, the fact that the duration of the notes is short means that the latency between the time when a note is fixated and the time when it is played might decrease. What guides musicians' ocular behavior therefore seems to be the time required to decode the score, with EHS (distance) adapting as a function of the temporal constraints.

Tempo is a factor that determines EHS variation and it seems important, even if it is not always actually imposed, at least to control for this factor *a posteriori*. Indeed, for experimental reasons, some studies do not impose a tempo in sight-reading tasks but instead measure the tempo chosen by the musician *a posteriori* (72). Furthermore, since spontaneous motor tempo (e.g., the preferred and natural pace to carry out isochronous motor actions) is relatively variable within and between subjects, for example it can differ depending on whether one is a musician or not (49) or depending on the time of the day (47), it is quite reasonable asking whether playing a score far from a musician’s spontaneous motor tempo would affect his EHS. That is why, when the eye-hand span is measured in terms of distance, we suggest taking account of the tempo chosen by the musician in order to calculate a ratio between EHS and tempo and contextualize the EHS measurement.

### Does training affect EHS? 

With training, and over the course of the sessions of musical practice devoted to preparing a piece of music, musicians increasingly use the hierarchical structure of the score in order to organize their execution of it. Thus, as they progress in their preparation of a piece, they start and stop their musical production at significant parts of the score: the start of phrases or structural markers (77). This allows them, on the one hand, to develop a structural representation of the score rather than a note-by-note representation and, on the other, to facilitate the memorization of the composition (2;8;17). Consequently, it is relevant to ask whether, as they internalize the musical structure of a score, musicians look further and further ahead relative to the point which they are currently performing. Even though it is difficult to talk about a sight-reading task when a score is learned (78), two studies have measured the change in EHS in musicians as they progress through the different stages involved in the learning of a score. In the study by Rosemann et al. (62), musicians had to sight-read a score and then train for 30 minutes before playing it again. The results showed that EHS (distance in musical units) did not increase significantly between the untrained playing of the score and the performance given after training. The authors proposed two interpretations to explain the absence of a training effect on EHS. Either 30 minutes was not long enough to allow the musicians to get to grips better with the score (floor effect), or the score was too simple and the musicians had a sufficiently high sight-reading level to achieve an optimum EHS the first time they played the piece (ceiling effect). Furthermore, Cara (2018) studied the effect of training on EHS modulated by expertise. The experiment consisted in repeating a sight-reading task four times with two minutes of training inserted between each trial. The results showed that only the less skilled participants benefited from the training. Their EHS increased to that of the level of the skilled musicians thanks to the repetitions. These results are consistent with the study conducted by Burman and Booth (42009), who presented musicians with a modified note detection task in which the effects of expertise on perceptual span weakened with training before disappearing altogether after 20 training sessions, with the least skilled musicians ultimately achieving the same performance as the most skilled ones. These results indicate that EHS and perceptual span can be a measure used to assess learning by showing how musicians internalize the structure of a score across training.

Too few studies have examined the effect of training on EHS. It would be interesting to propose experimental designs in which the complexity of the scores differs and in which the length of training is modulated in order to measure whether internalization of the structure of the score during learning affects EHS.

### Does the type of instrument influence EHS?

Among the studies that have used EHS-like measurements in musicians, one has investigated the eye-stroke span (ESS) in xylophonists (44) and another has examined the eye-time span (ETS) in singers (12). The first measured the latency between the time when the xylophonist fixated a key and the time he or she played it. It should be noted that the musicians did not have the scores in front of them since they already knew the music. The results showed that even though there was no music to read, the ESS was approximately 2 to 3 notes on average, thus suggesting that the musicians still have to anticipate in order to plan the motor activity involved in striking the keys. Finally, in the study by Chitalkina et al. (12), the authors compared the sight-reading ETS of pianists with the sight-reading ETS in singers. In this study, the singers and pianists had to read scores in different tonalities (complex: B major / less complex: C major) in which one bar was incongruent (step-down division). The results revealed an interaction between the performance modality (singing and piano) and tonality (B/C), thus indicating that when the pianists fixated the second part of the incongruent bar, they had a higher ETS than the singers in the complex tonality, whereas this difference was small with the simple tonality. The authors interpreted this difference as being the effect of a twofold difficulty (complex tonality, incongruent division) on motor planning, which was greater in the case of piano-playing than singing. Even though singing also requires motor production, this type of experimental design makes it possible to measure the proportion of processing attributable to motor planning in musicians' EHS. Although these studies are still exploratory, they show that each instrument used in studies might be taken into account to compare musicians’ EHS and they pave the way for questions relating to the difference in the cognitive demands associated with inter-instrument motor planning. For example, it would be interesting to measure the effect of the type of instrument on EHS depending on whether they require the two hands to be coordinated for the execution of similar movements (e.g., keyboard instruments, flute) or of different movements (e.g., violin, cello).

## Conclusion

This review of the literature relates to the measurement of EHS in music-reading tasks, either in sight-reading tasks or in tasks that involve music performance (i.e., 44). The aim is to give the scientific community the key information needed in order to understand this field and indicate avenues for research. The summary of the methodologies and theories relating to the measurement of EHS makes it clear why so few studies (15) have been devoted to measuring EHS. The task is difficult and considerable scientific rigor must be exercised if the results are not to be unusable. Nevertheless, the existing works make it possible to identify interesting theoretical advances and new areas of exploration to which this field of study can turn.

**Figure 8.**
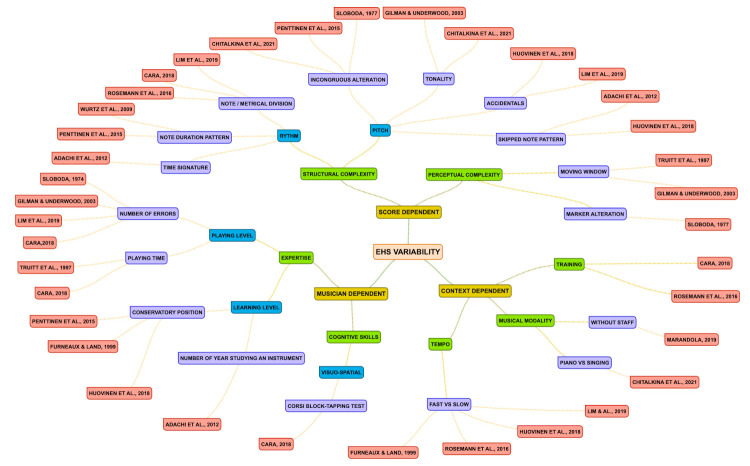
Mind-mapping representation of eye-hand span variability. This figure can be read from the inside out. Starting from the central box “EHS variability”, it is possible to follow a path to each study through the type of factor and methodology used. For example, among the studies that varied a score-dependent factor, Lim et al.'s (42) manipulated the structural complexity of the score by varying its pitch (in this case the number of accidentals in the score). A single study may have manipulated several factors.

First of all, and contrary to some teaching methods which recommend looking as far ahead as possible in the score (3; 21), the EHS on the score is actually quite small and the eye only rarely fixates a bar ahead of what is currently being played, even among the most skilled musicians (72). Nevertheless, even if EHS is quite small, studies show that it depends on a number of different factors (see *Figure 8*). EHS is sensitive to both top-down processes, such as musical expertise, and bottom-up processes, such as the difficulty engendered by the score and the context in which the piece is played (i.e., tempo, training, instrument; see *Figure 8*). Indeed, the more skilled musicians are, the greater their EHS is (1;7;22;23;28;42;51;66; 72), and the more complex a score is, the shorter the EHS, even in the case of skilled musicians (1; 7;23;42;51;62;67;72; 80). Finally, the vital factor appears to be the ability of musicians to modify their EHS during sight-reading (12;28;42). In effect, the musicians with the best sight-reading performances seem to have an EHS which is inversely proportional to the complexity of the score, seemingly indicating that skilled musicians have a high level of perceptual flexibility (42). At the same time, the local analysis of musicians' behavior while playing a score seems to indicate that the presence of a complexity in the parafoveal region can attract the next eye fixation in both temporal (the eye fixation arrives earlier at or near to a complexity) and spatial terms (the incoming eye saccade at or near to a complexity is greater; 12; 28). Thus, EHS seems to vary within one and the same score as a function of the local complexity. This confirms the idea that skilled musicians adapt their EHS during sight reading and suggests that measurements of EHS should also take account of the intra-score context.

Furthermore, this review of the state of the art makes it possible to elaborate a methodological view of the literature on eye movements during sight reading. This review makes no claim to being exhaustive in naming all the factors that affect EHS, but simply those that have been studied in the literature. Firstly, we want to point out the differences in the criteria used to define expertise (learning vs. playing level). To optimize the study of what it is that characterizes a good EHS eye movement study, it seems appropriate to take account of playing level during the task in addition to learning level in order to be certain that the category of the best sight readers have indeed used strategies that have brought about better performance. Secondly, a consideration of all the literature available on EHS seems to indicate that the definition of score complexity is variable. Since the complexity of a score can be measured in terms of different indicators, it would be interesting to put forward a model of the change in EHS as a function of the type of difficulty (e.g., pitch, rhythm), its level (e.g., note that is unpredictable, difficult to produce), salience (e.g., accidentals, sharps or flats in the key signature) or its prolonged or temporary nature (e.g., entire score, one metrical division, one note) in order to study the way the eye is attracted and slows down while decoding a score as a function of these factors. Ultimately, it will be possible to discriminate musical material by means of an index that is based on these criteria.

Finally, this review shows that, independently of the musician's expertise and the complexity of the score, the context in which the music-reading is performed can influence EHS. Tempo is a factor that determines the size of EHS: On the one hand, when EHS is measured as a function of musical units, it seems to increase the faster the tempo is (28;62), indicating that musicians need to move their gaze further on in the score in order to anticipate more notes and plan their motor actions. At the same time, when EHS is measured in absolute time, it seems to fall the faster the tempo is (22;62), indicating that although musicians fixate their gaze further on in the score when the tempo increases, the latency between the time when a note is fixated and the time when it is played might decrease. It therefore seems to be the time required to decode the score (more or less 1000 ms) that guides musicians' eye-movement behaviors, with EHS (distance) adapting as a function of the temporal constraints of the score. This is why it seems to be necessary to take account of and control for the tempo chosen by musicians in order to establish a relation between their EHS and the time taken to play the score. Furthermore, two studies have examined the change in EHS with musical practice, including one study which has shown the tendency of the EHS of less skilled musicians to increase after learning the score (7). It would appear interesting to perform a longitudinal study in which the training effect is measured over a longer period and with scores of different complexities in order to measure the ability to internalize the musical structure during learning on the basis of EHS.

In a desire to bring about the development of increasingly fine-grained models of changes in behavior during sight reading, this review shows that EHS is a flagship measure for the evaluation of music reading because it represents the ability to adapt and make use of strategies in order to overcome the difficulties present in a score. In the long term, the combination of this measure with other measures obtained from eye-movement analyses could make it possible to come to a fine-grained definition of what it is that characterizes effective music-reading strategies.

## Ethics and Conflict of Interest

The author(s) declare(s) that the contents of the article are in agreement with the ethics described in http://biblio.unibe.ch/portale/elibrary/BOP/jemr/ethics.html and that there is no conflict of interest regarding the publication of this paper.

## Acknowledgement

This work was supported by the French Agence Nationale de la Recherche (ANR JCJC MUREA project, grant ANR-18-CE38-0006-01).
